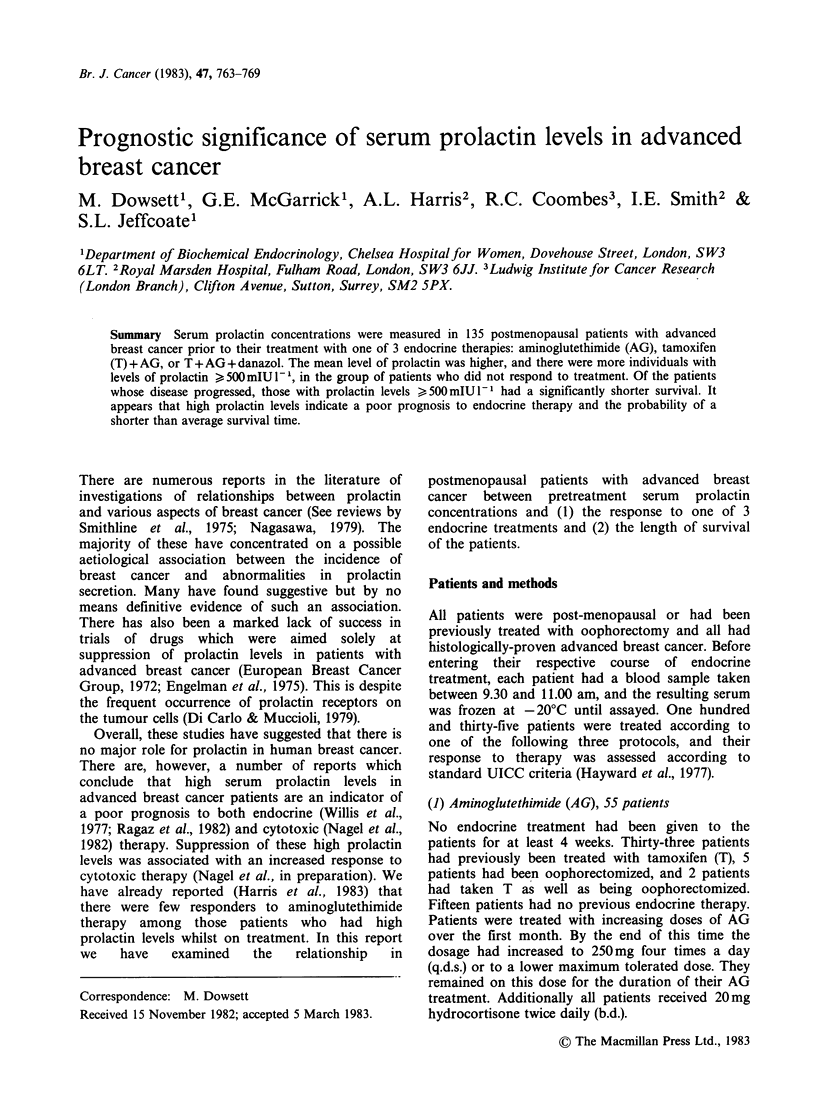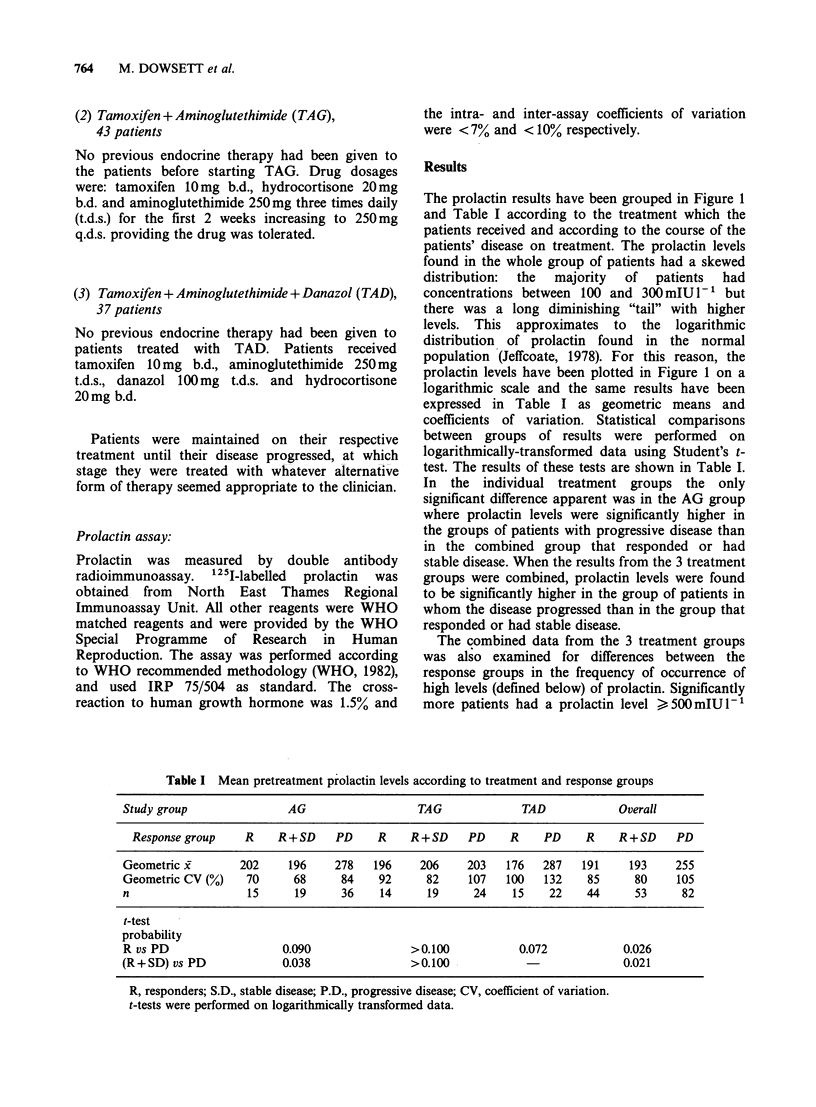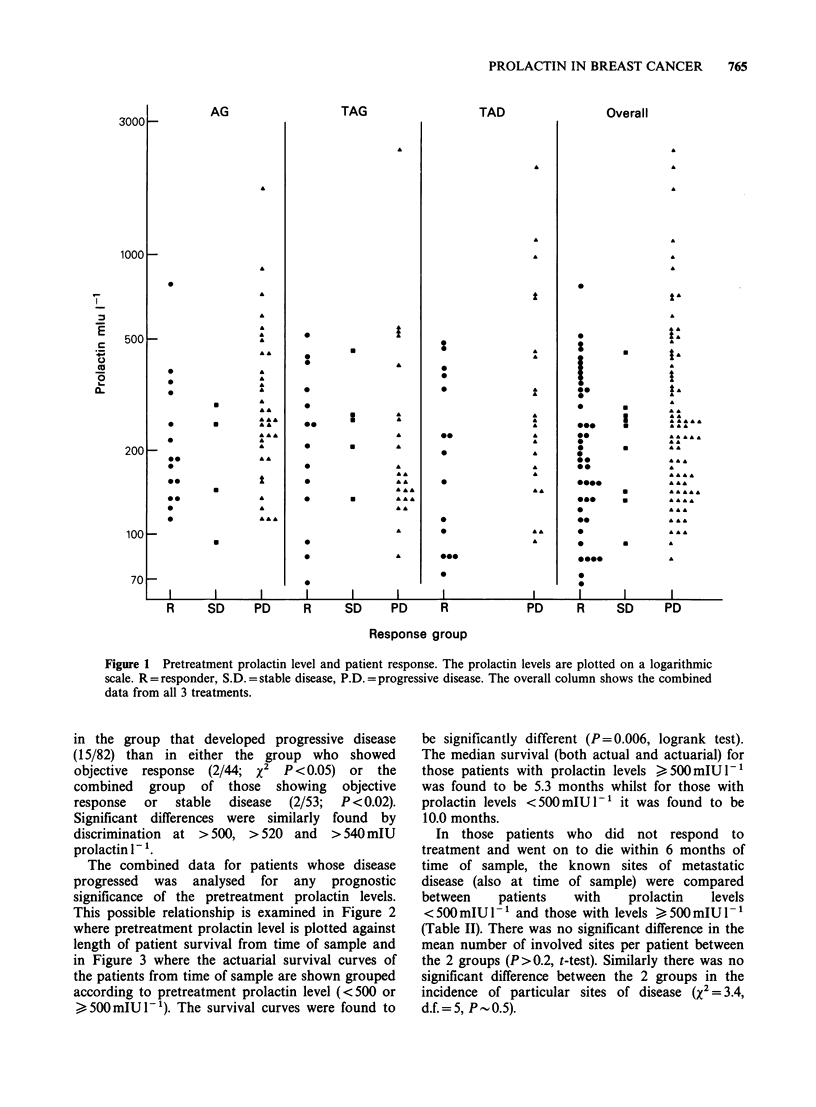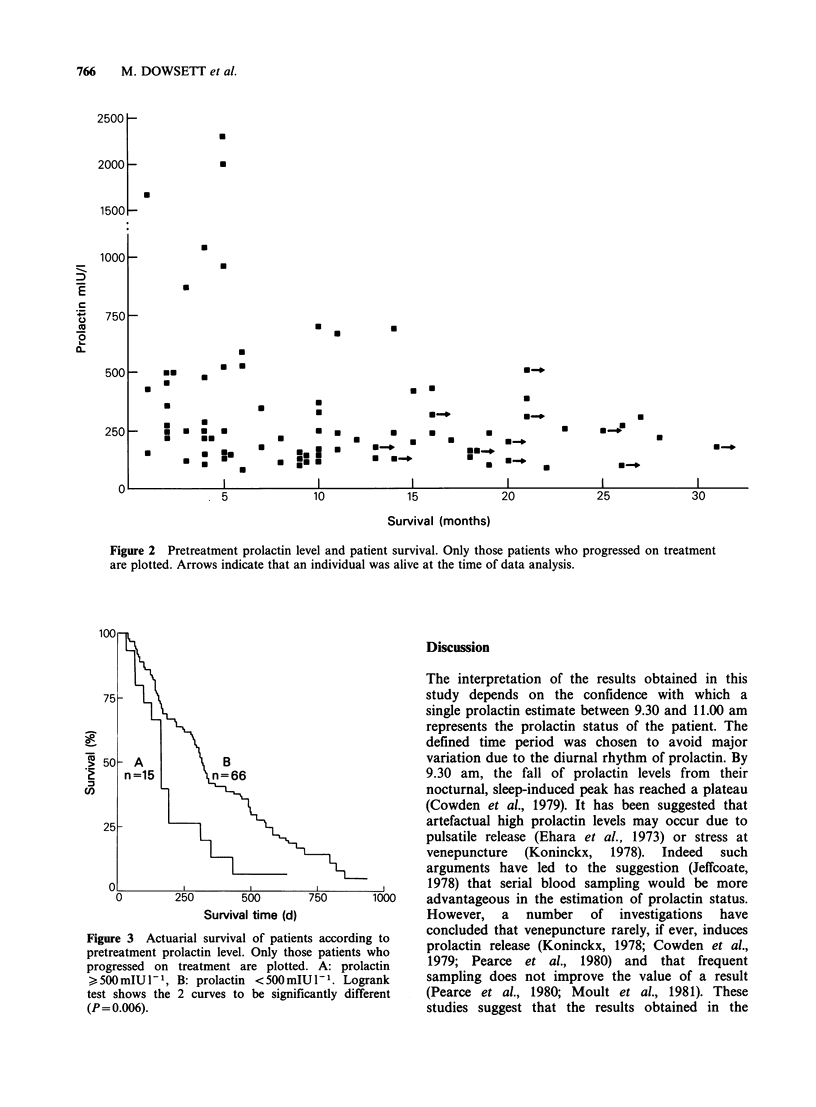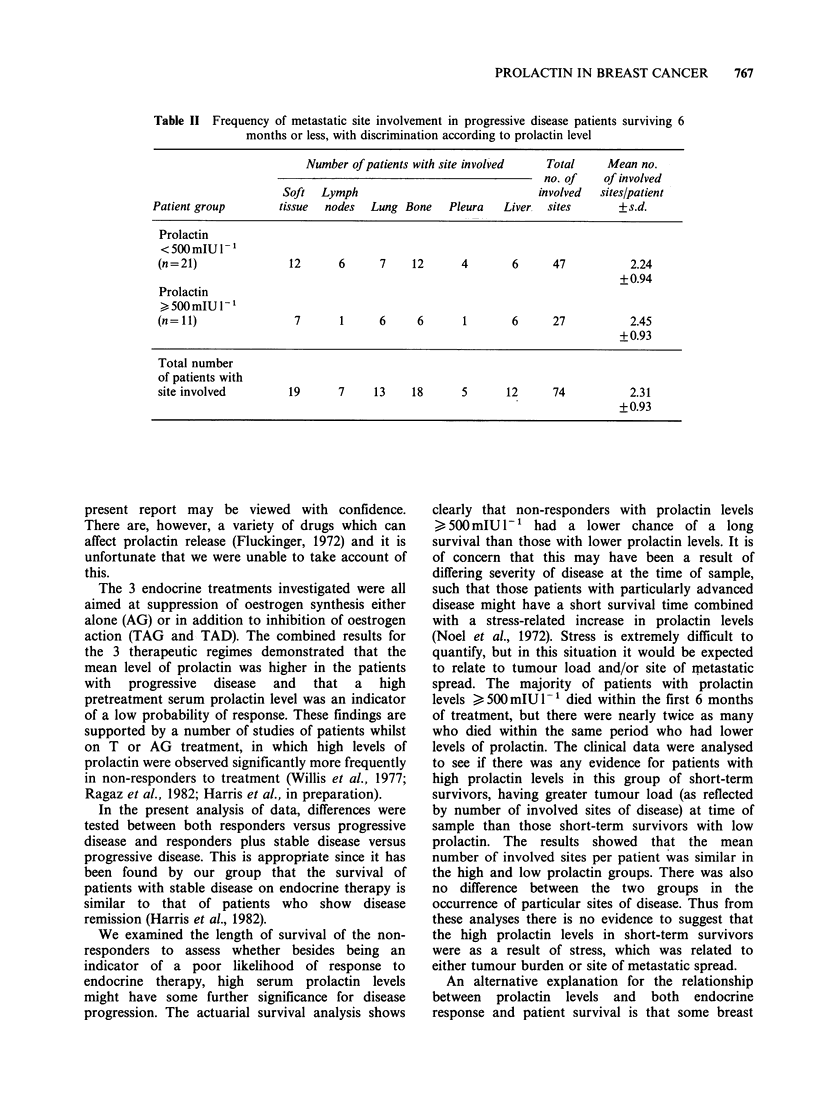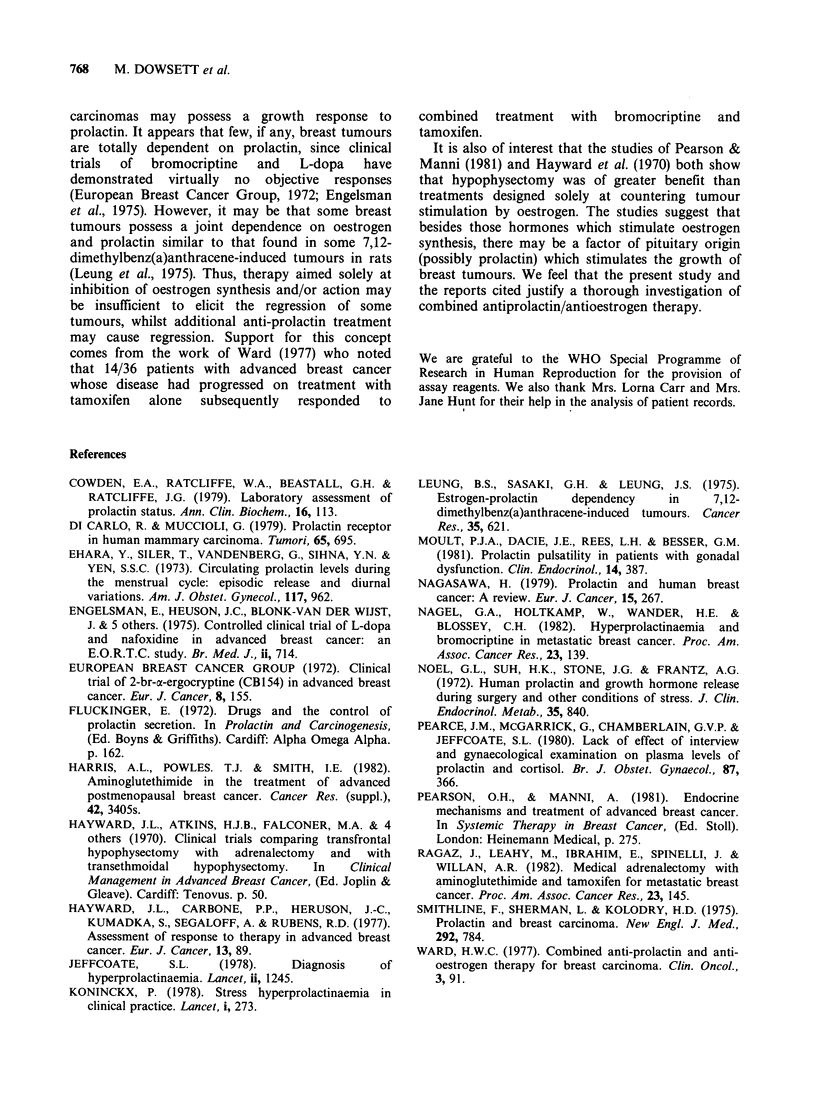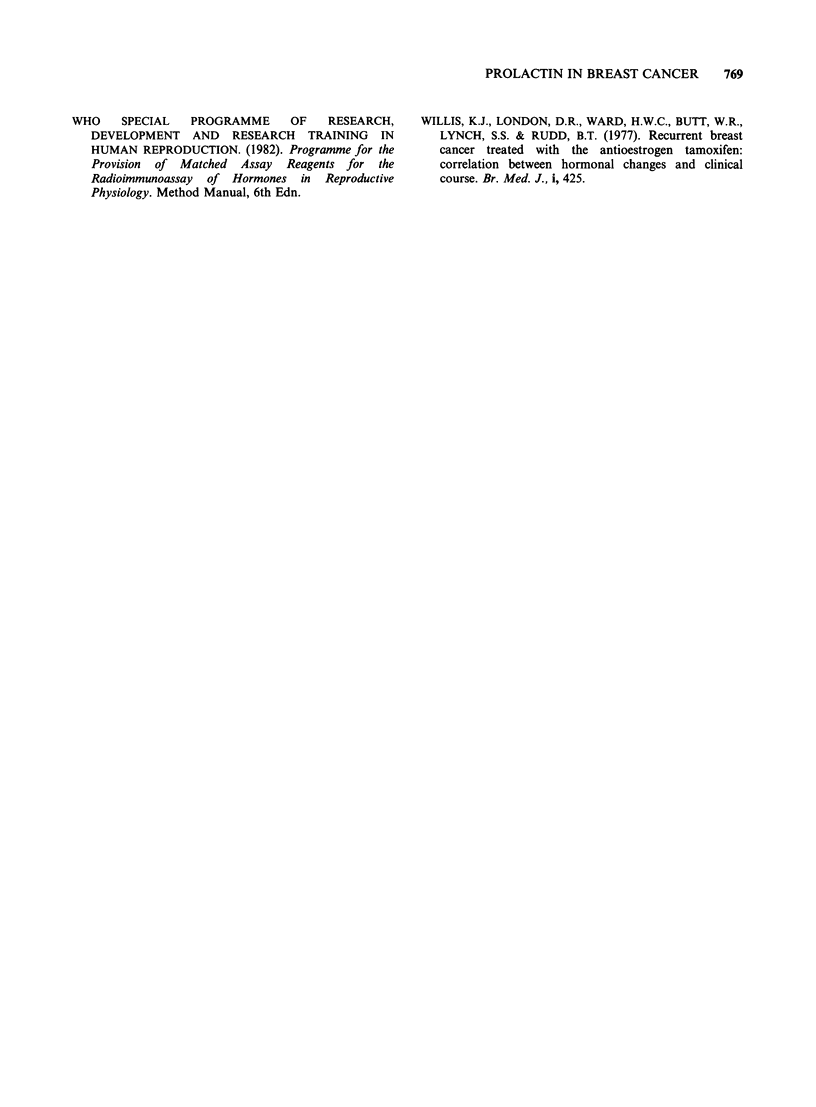# Prognostic significance of serum prolactin levels in advanced breast cancer.

**DOI:** 10.1038/bjc.1983.129

**Published:** 1983-06

**Authors:** M. Dowsett, G. E. McGarrick, A. L. Harris, R. C. Coombes, I. E. Smith, S. L. Jeffcoate

## Abstract

Serum prolactin concentrations were measured in 135 postmenopausal patients with advanced breast cancer prior to their treatment with one of 3 endocrine therapies: aminoglutethimide (AG), tamoxifen (T) + AG, or T + AG + danazol. The mean level of prolactin was higher, and there were more individuals with levels of prolactin greater than or equal to 500 mIUl-1, in the group of patients who did not respond to treatment. Of the patients whose disease progressed, those with prolactin levels greater than or equal to 500 mIUl-1 had a significantly shorter survival. It appears that high prolactin levels indicate a poor prognosis to endocrine therapy and the probability of a shorter than average survival time.


Br. J. Cancer (1983), 47, 763–769

### Prognostic significance of serum prolactin levels in advanced breast cancer

M. Dowsett[1], G.E. McGarrick[1], A.L. Harris[2], R.C. Coombes[3], I.E. Smith[2] & S.L. Jeffcoate[1]

[1]*Department of Biochemical Endocrinology, Chelsea Hospital for Women, Dovehouse Street, London, SW3 6LT.* [2]*Royal Marsden Hospital, Fulham Road, London, SW3 6JJ.* [3]*Ludwig Institute for Cancer Research (London Branch), Clifton Avenue, Sutton, Surrey, SM2 5PX.*

**Summary** Serum prolactin concentrations were measured in 135 postmenopausal patients with advanced breast cancer prior to their treatment with one of 3 endocrine therapies: aminoglutethimide (AG), tamoxifen (T)+AG, or T+AG+danazol. The mean level of prolactin was higher, and there were more individuals with levels of prolactin $\geqslant 500\,\text{mIU}\,\text{l}^{-1}$, in the group of patients who did not respond to treatment. Of the patients whose disease progressed, those with prolactin levels $\geqslant 500\,\text{mIU}\,\text{l}^{-1}$ had a significantly shorter survival. It appears that high prolactin levels indicate a poor prognosis to endocrine therapy and the probability of a shorter than average survival time.

There are numerous reports in the literature of investigations of relationships between prolactin and various aspects of breast cancer (See reviews by Smithline *et al.*, 1975; Nagasawa, 1979). The majority of these have concentrated on a possible aetiological association between the incidence of breast cancer and abnormalities in prolactin secretion. Many have found suggestive but by no means definitive evidence of such an association. There has also been a marked lack of success in trials of drugs which were aimed solely at suppression of prolactin levels in patients with advanced breast cancer (European Breast Cancer Group, 1972; Engelman *et al.*, 1975). This is despite the frequent occurrence of prolactin receptors on the tumour cells (Di Carlo & Muccioli, 1979).

Overall, these studies have suggested that there is no major role for prolactin in human breast cancer. There are, however, a number of reports which conclude that high serum prolactin levels in advanced breast cancer patients are an indicator of a poor prognosis to both endocrine (Willis *et al.*, 1977; Ragaz *et al.*, 1982) and cytotoxic (Nagel *et al.*, 1982) therapy. Suppression of these high prolactin levels was associated with an increased response to cytotoxic therapy (Nagel *et al.*, in preparation). We have already reported (Harris *et al.*, 1983) that there were few responders to aminoglutethimide therapy among those patients who had high prolactin levels whilst on treatment. In this report we have examined the relationship in postmenopausal patients with advanced breast cancer between pretreatment serum prolactin concentrations and (1) the response to one of 3 endocrine treatments and (2) the length of survival of the patients.

Correspondence: M. Dowsett

Received 15 November 1982; accepted 5 March 1983.

### Patients and methods

All patients were post-menopausal or had been previously treated with oophorectomy and all had histologically-proven advanced breast cancer. Before entering their respective course of endocrine treatment, each patient had a blood sample taken between 9.30 and 11.00 am, and the resulting serum was frozen at −20°C until assayed. One hundred and thirty-five patients were treated according to one of the following three protocols, and their response to therapy was assessed according to standard UICC criteria (Hayward *et al.*, 1977).

*(1) Aminoglutethimide (AG), 55 patients*

No endocrine treatment had been given to the patients for at least 4 weeks. Thirty-three patients had previously been treated with tamoxifen (T), 5 patients had been oophorectomized, and 2 patients had taken T as well as being oophorectomized. Fifteen patients had no previous endocrine therapy. Patients were treated with increasing doses of AG over the first month. By the end of this time the dosage had increased to 250 mg four times a day (q.d.s.) or to a lower maximum tolerated dose. They remained on this dose for the duration of their AG treatment. Additionally all patients received 20 mg hydrocortisone twice daily (b.d.).

© The Macmillan Press Ltd., 1983

(2) *Tamoxifen + Aminoglutethimide (TAG), 43 patients*

No previous endocrine therapy had been given to the patients before starting TAG. Drug dosages were: tamoxifen 10 mg b.d., hydrocortisone 20 mg b.d. and aminoglutethimide 250 mg three times daily (t.d.s.) for the first 2 weeks increasing to 250 mg q.d.s. providing the drug was tolerated.

(3) *Tamoxifen + Aminoglutethimide + Danazol (TAD), 37 patients*

No previous endocrine therapy had been given to patients treated with TAD. Patients received tamoxifen 10 mg b.d., aminoglutethimide 250 mg t.d.s., danazol 100 mg t.d.s. and hydrocortisone 20 mg b.d.

Patients were maintained on their respective treatment until their disease progressed, at which stage they were treated with whatever alternative form of therapy seemed appropriate to the clinician.

*Prolactin assay:*

Prolactin was measured by double antibody radioimmunoassay. $^{125}$I-labelled prolactin was obtained from North East Thames Regional Immunoassay Unit. All other reagents were WHO matched reagents and were provided by the WHO Special Programme of Research in Human Reproduction. The assay was performed according to WHO recommended methodology (WHO, 1982), and used IRP 75/504 as standard. The cross-reaction to human growth hormone was 1.5% and the intra- and inter-assay coefficients of variation were $<7\%$ and $<10\%$ respectively.

### Results

The prolactin results have been grouped in Figure 1 and Table I according to the treatment which the patients received and according to the course of the patients' disease on treatment. The prolactin levels found in the whole group of patients had a skewed distribution: the majority of patients had concentrations between 100 and 300 mIU $l^{-1}$ but there was a long diminishing "tail" with higher levels. This approximates to the logarithmic distribution of prolactin found in the normal population (Jeffcoate, 1978). For this reason, the prolactin levels have been plotted in Figure 1 on a logarithmic scale and the same results have been expressed in Table I as geometric means and coefficients of variation. Statistical comparisons between groups of results were performed on logarithmically-transformed data using Student's $t$-test. The results of these tests are shown in Table I. In the individual treatment groups the only significant difference apparent was in the AG group where prolactin levels were significantly higher in the groups of patients with progressive disease than in the combined group that responded or had stable disease. When the results from the 3 treatment groups were combined, prolactin levels were found to be significantly higher in the group of patients in whom the disease progressed than in the group that responded or had stable disease.

The combined data from the 3 treatment groups was also examined for differences between the response groups in the frequency of occurrence of high levels (defined below) of prolactin. Significantly more patients had a prolactin level $\geqslant 500$ mIU $l^{-1}$

**Table I** Mean pretreatment prolactin levels according to treatment and response groups

| *Study group* | *AG* | | | *TAG* | | | *TAD* | | *Overall* | | |
|---|---|---|---|---|---|---|---|---|---|---|---|
| *Response group* | *R* | *R+SD* | *PD* | *R* | *R+SD* | *PD* | *R* | *PD* | *R* | *R+SD* | *PD* |
| Geometric $\bar{x}$ | 202 | 196 | 278 | 196 | 206 | 203 | 176 | 287 | 191 | 193 | 255 |
| Geometric CV (%) | 70 | 68 | 84 | 92 | 82 | 107 | 100 | 132 | 85 | 80 | 105 |
| $n$ | 15 | 19 | 36 | 14 | 19 | 24 | 15 | 22 | 44 | 53 | 82 |
| $t$-test probability | | | | | | | | | | | |
| R *vs* PD | | 0.090 | | | >0.100 | | 0.072 | | | 0.026 | |
| (R+SD) *vs* PD | | 0.038 | | | >0.100 | | — | | | 0.021 | |

R, responders; S.D., stable disease; P.D., progressive disease; CV, coefficient of variation.
$t$-tests were performed on logarithmically transformed data.

**Figure 1** Pretreatment prolactin level and patient response. The prolactin levels are plotted on a logarithmic scale. R = responder, S.D. = stable disease, P.D. = progressive disease. The overall column shows the combined data from all 3 treatments.

in the group that developed progressive disease (15/82) than in either the group who showed objective response (2/44; $\chi^2$ $P<0.05$) or the combined group of those showing objective response or stable disease (2/53; $P<0.02$). Significant differences were similarly found by discrimination at $>500$, $>520$ and $>540$ mIU prolactin $l^{-1}$.

The combined data for patients whose disease progressed was analysed for any prognostic significance of the pretreatment prolactin levels. This possible relationship is examined in Figure 2 where pretreatment prolactin level is plotted against length of patient survival from time of sample and in Figure 3 where the actuarial survival curves of the patients from time of sample are shown grouped according to pretreatment prolactin level ($<500$ or $\geqslant 500$ mIU $l^{-1}$). The survival curves were found to be significantly different ($P=0.006$, logrank test). The median survival (both actual and actuarial) for those patients with prolactin levels $\geqslant 500$ mIU $l^{-1}$ was found to be 5.3 months whilst for those with prolactin levels $<500$ mIU $l^{-1}$ it was found to be 10.0 months.

In those patients who did not respond to treatment and went on to die within 6 months of time of sample, the known sites of metastatic disease (also at time of sample) were compared between patients with prolactin levels $<500$ mIU $l^{-1}$ and those with levels $\geqslant 500$ mIU $l^{-1}$ (Table II). There was no significant difference in the mean number of involved sites per patient between the 2 groups ($P>0.2$, $t$-test). Similarly there was no significant difference between the 2 groups in the incidence of particular sites of disease ($\chi^2=3.4$, d.f. = 5, $P\sim0.5$).

**Figure 2** Pretreatment prolactin level and patient survival. Only those patients who progressed on treatment are plotted. Arrows indicate that an individual was alive at the time of data analysis.

**Figure 3** Actuarial survival of patients according to pretreatment prolactin level. Only those patients who progressed on treatment are plotted. A: prolactin $\geqslant 500\,mIU\,l^{-1}$, B: prolactin $<500\,mIU\,l^{-1}$. Logrank test shows the 2 curves to be significantly different ($P=0.006$).

### Discussion

The interpretation of the results obtained in this study depends on the confidence with which a single prolactin estimate between 9.30 and 11.00 am represents the prolactin status of the patient. The defined time period was chosen to avoid major variation due to the diurnal rhythm of prolactin. By 9.30 am, the fall of prolactin levels from their nocturnal, sleep-induced peak has reached a plateau (Cowden *et al.*, 1979). It has been suggested that artefactual high prolactin levels may occur due to pulsatile release (Ehara *et al.*, 1973) or stress at venepuncture (Koninckx, 1978). Indeed such arguments have led to the suggestion (Jeffcoate, 1978) that serial blood sampling would be more advantageous in the estimation of prolactin status. However, a number of investigations have concluded that venepuncture rarely, if ever, induces prolactin release (Koninckx, 1978; Cowden *et al.*, 1979; Pearce *et al.*, 1980) and that frequent sampling does not improve the value of a result (Pearce *et al.*, 1980; Moult *et al.*, 1981). These studies suggest that the results obtained in the

**Table II** Frequency of metastatic site involvement in progressive disease patients surviving 6 months or less, with discrimination according to prolactin level

| *Patient group* | *Number of patients with site involved* *Soft tissue* | *Lymph nodes* | *Lung* | *Bone* | *Pleura* | *Liver* | *Total no. of involved sites* | *Mean no. of involved sites/patient* $\pm$*s.d.* |
|---|---|---|---|---|---|---|---|---|
| Prolactin $<500\,mIU\,l^{-1}$ ($n=21$) | 12 | 6 | 7 | 12 | 4 | 6 | 47 | 2.24 $\pm 0.94$ |
| Prolactin $\geqslant 500\,mIU\,l^{-1}$ ($n=11$) | 7 | 1 | 6 | 6 | 1 | 6 | 27 | 2.45 $\pm 0.93$ |
| Total number of patients with site involved | 19 | 7 | 13 | 18 | 5 | 12 | 74 | 2.31 $\pm 0.93$ |

present report may be viewed with confidence. There are, however, a variety of drugs which can affect prolactin release (Fluckinger, 1972) and it is unfortunate that we were unable to take account of this.

The 3 endocrine treatments investigated were all aimed at suppression of oestrogen synthesis either alone (AG) or in addition to inhibition of oestrogen action (TAG and TAD). The combined results for the 3 therapeutic regimes demonstrated that the mean level of prolactin was higher in the patients with progressive disease and that a high pretreatment serum prolactin level was an indicator of a low probability of response. These findings are supported by a number of studies of patients whilst on T or AG treatment, in which high levels of prolactin were observed significantly more frequently in non-responders to treatment (Willis *et al.*, 1977; Ragaz *et al.*, 1982; Harris *et al.*, in preparation).

In the present analysis of data, differences were tested between both responders versus progressive disease and responders plus stable disease versus progressive disease. This is appropriate since it has been found by our group that the survival of patients with stable disease on endocrine therapy is similar to that of patients who show disease remission (Harris *et al.*, 1982).

We examined the length of survival of the non-responders to assess whether besides being an indicator of a poor likelihood of response to endocrine therapy, high serum prolactin levels might have some further significance for disease progression. The actuarial survival analysis shows clearly that non-responders with prolactin levels $\geqslant 500\,mIU\,l^{-1}$ had a lower chance of a long survival than those with lower prolactin levels. It is of concern that this may have been a result of differing severity of disease at the time of sample, such that those patients with particularly advanced disease might have a short survival time combined with a stress-related increase in prolactin levels (Noel *et al.*, 1972). Stress is extremely difficult to quantify, but in this situation it would be expected to relate to tumour load and/or site of metastatic spread. The majority of patients with prolactin levels $\geqslant 500\,mIU\,l^{-1}$ died within the first 6 months of treatment, but there were nearly twice as many who died within the same period who had lower levels of prolactin. The clinical data were analysed to see if there was any evidence for patients with high prolactin levels in this group of short-term survivors, having greater tumour load (as reflected by number of involved sites of disease) at time of sample than those short-term survivors with low prolactin. The results showed that the mean number of involved sites per patient was similar in the high and low prolactin groups. There was also no difference between the two groups in the occurrence of particular sites of disease. Thus from these analyses there is no evidence to suggest that the high prolactin levels in short-term survivors were as a result of stress, which was related to either tumour burden or site of metastatic spread.

An alternative explanation for the relationship between prolactin levels and both endocrine response and patient survival is that some breast carcinomas may possess a growth response to prolactin. It appears that few, if any, breast tumours are totally dependent on prolactin, since clinical trials of bromocriptine and L-dopa have demonstrated virtually no objective responses (European Breast Cancer Group, 1972; Engelsman et al., 1975). However, it may be that some breast tumours possess a joint dependence on oestrogen and prolactin similar to that found in some 7,12-dimethylbenz(a)anthracene-induced tumours in rats (Leung et al., 1975). Thus, therapy aimed solely at inhibition of oestrogen synthesis and/or action may be insufficient to elicit the regression of some tumours, whilst additional anti-prolactin treatment may cause regression. Support for this concept comes from the work of Ward (1977) who noted that 14/36 patients with advanced breast cancer whose disease had progressed on treatment with tamoxifen alone subsequently responded to combined treatment with bromocriptine and tamoxifen.

It is also of interest that the studies of Pearson & Manni (1981) and Hayward et al. (1970) both show that hypophysectomy was of greater benefit than treatments designed solely at countering tumour stimulation by oestrogen. The studies suggest that besides those hormones which stimulate oestrogen synthesis, there may be a factor of pituitary origin (possibly prolactin) which stimulates the growth of breast tumours. We feel that the present study and the reports cited justify a thorough investigation of combined antiprolactin/antioestrogen therapy.

We are grateful to the WHO Special Programme of Research in Human Reproduction for the provision of assay reagents. We also thank Mrs. Lorna Carr and Mrs. Jane Hunt for their help in the analysis of patient records.

### References

COWDEN, E.A., RATCLIFFE, W.A., BEASTALL, G.H. & RATCLIFFE, J.G. (1979). Laboratory assessment of prolactin status. *Ann. Clin. Biochem.*, **16**, 113.

DI CARLO, R. & MUCCIOLI, G. (1979). Prolactin receptor in human mammary carcinoma. *Tumori*, **65**, 695.

EHARA, Y., SILER, T., VANDENBERG, G., SIHNA, Y.N. & YEN, S.S.C. (1973). Circulating prolactin levels during the menstrual cycle: episodic release and diurnal variations. *Am. J. Obstet. Gynecol.*, **117**, 962.

ENGELSMAN, E., HEUSON, J.C., BLONK-VAN DER WIJST, J. & 5 others. (1975). Controlled clinical trial of L-dopa and nafoxidine in advanced breast cancer: an E.O.R.T.C. study. *Br. Med. J.*, **ii**, 714.

EUROPEAN BREAST CANCER GROUP (1972). Clinical trial of 2-br-α-ergocryptine (CB154) in advanced breast cancer. *Eur. J. Cancer*, **8**, 155.

FLUCKINGER, E. (1972). Drugs and the control of prolactin secretion. In *Prolactin and Carcinogenesis*, (Ed. Boyns & Griffiths). Cardiff: Alpha Omega Alpha. p. 162.

HARRIS, A.L., POWLES. T.J. & SMITH, I.E. (1982). Aminoglutethimide in the treatment of advanced postmenopausal breast cancer. *Cancer Res.* (suppl.), **42**, 3405s.

HAYWARD, J.L., ATKINS, H.J.B., FALCONER, M.A. & 4 others (1970). Clinical trials comparing transfrontal hypophysectomy with adrenalectomy and with transethmoidal hypophysectomy. In *Clinical Management in Advanced Breast Cancer*, (Ed. Joplin & Gleave). Cardiff: Tenovus. p. 50.

HAYWARD, J.L., CARBONE, P.P., HERUSON, J.-C., KUMADKA, S., SEGALOFF, A. & RUBENS, R.D. (1977). Assessment of response to therapy in advanced breast cancer. *Eur. J. Cancer*, **13**, 89.

JEFFCOATE, S.L. (1978). Diagnosis of hyperprolactinaemia. *Lancet*, **ii**, 1245.

KONINCKX, P. (1978). Stress hyperprolactinaemia in clinical practice. *Lancet*, **i**, 273.

LEUNG, B.S., SASAKI, G.H. & LEUNG, J.S. (1975). Estrogen-prolactin dependency in 7,12-dimethylbenz(a)anthracene-induced tumours. *Cancer Res.*, **35**, 621.

MOULT, P.J.A., DACIE, J.E., REES, L.H. & BESSER, G.M. (1981). Prolactin pulsatility in patients with gonadal dysfunction. *Clin. Endocrinol.*, **14**, 387.

NAGASAWA, H. (1979). Prolactin and human breast cancer: A review. *Eur. J. Cancer*, **15**, 267.

NAGEL, G.A., HOLTKAMP, W., WANDER, H.E. & BLOSSEY, C.H. (1982). Hyperprolactinaemia and bromocriptine in metastatic breast cancer. *Proc. Am. Assoc. Cancer Res.*, **23**, 139.

NOEL, G.L., SUH, H.K., STONE, J.G. & FRANTZ, A.G. (1972). Human prolactin and growth hormone release during surgery and other conditions of stress. *J. Clin. Endocrinol. Metab.*, **35**, 840.

PEARCE, J.M., MCGARRICK, G., CHAMBERLAIN, G.V.P. & JEFFCOATE, S.L. (1980). Lack of effect of interview and gynaecological examination on plasma levels of prolactin and cortisol. *Br. J. Obstet. Gynaecol.*, **87**, 366.

PEARSON, O.H., & MANNI, A. (1981). Endocrine mechanisms and treatment of advanced breast cancer. In *Systemic Therapy in Breast Cancer*, (Ed. Stoll). London: Heinemann Medical, p. 275.

RAGAZ, J., LEAHY, M., IBRAHIM, E., SPINELLI, J. & WILLAN, A.R. (1982). Medical adrenalectomy with aminoglutethimide and tamoxifen for metastatic breast cancer. *Proc. Am. Assoc. Cancer Res.*, **23**, 145.

SMITHLINE, F., SHERMAN, L. & KOLODRY, H.D. (1975). Prolactin and breast carcinoma. *New Engl. J. Med.*, **292**, 784.

WARD, H.W.C. (1977). Combined anti-prolactin and anti-oestrogen therapy for breast carcinoma. *Clin. Oncol.*, **3**, 91.

WHO SPECIAL PROGRAMME OF RESEARCH, DEVELOPMENT AND RESEARCH TRAINING IN HUMAN REPRODUCTION. (1982). *Programme for the Provision of Matched Assay Reagents for the Radioimmunoassay of Hormones in Reproductive Physiology*. Method Manual, 6th Edn.

WILLIS, K.J., LONDON, D.R., WARD, H.W.C., BUTT, W.R., LYNCH, S.S. & RUDD, B.T. (1977). Recurrent breast cancer treated with the antioestrogen tamoxifen: correlation between hormonal changes and clinical course. *Br. Med. J.*, **i**, 425.